# Emerging roles and mechanisms of miR-206 in human disorders: a comprehensive review

**DOI:** 10.1186/s12935-022-02833-2

**Published:** 2022-12-17

**Authors:** Sheyda Khalilian, Seyedeh Zahra Hosseini Imani, Soudeh Ghafouri-Fard

**Affiliations:** 1 Student Research Committee, School of Medicine, Shahid Beheshti University of Medical Sciences, Tehran, Iran; 2 Department of Medical Genetics, School of Medicine, Shahid Beheshti University of Medical Sciences, Tehran, Iran; 3 USERN Office, Shahid Beheshti University of Medical Sciences, Tehran, Iran; 4 Division of Genetics, Department of Cell and Molecular Biology and Microbiology, Faculty of Biological Sciences and Technologies, University of Isfahan, Esfahān, Iran

**Keywords:** miR-206, Cancer, Biomarkers, Noncoding RNA, microRNAs

## Abstract

As a member of the miR-1 family, miR-206 is located between *IL-17* and *PKHD1* genes in human. This miRNA has been shown to be involved in the pathogenic processes in a variety of human disorders including cancers, amyotrophic lateral sclerosis, Alzheimer’s disease, atherosclerosis, bronchopulmonary dysplasia, coronary artery disease, chronic obstructive pulmonary disease, epilepsy, nonalcoholic fatty liver disease, Hirschsprung disease, muscular dystrophies, pulmonary arterial hypertension, sepsis and ulcerative colitis. In the current review, we summarize the role of miR-206 in both malignant and non-malignant situations and explain its possible therapeutic implications.

## Introduction

MicroRNAs (miRNAs) are a group of naturally happening short non-coding RNAs with 21 to 22 nucleotide long. These transcripts contribute to post-transcriptional silencing of target genes [1, 2]. A single miRNA can affect expression of thousands of mRNAs and their target genes [3, 4]. Universally, miRNAs interact with 3′UTR to inhibit translation or degrade target transcripts. The critical seed region of miRNAs is located in the nucleotides 2–7 of their 5′UTR [5, 6]. miRNAs participate in several critical regulatory functions associated with cell growth, developmental processes and differentiation. Dysregulation of miRNAs is associated with a wide array of human pathway pathologies, especially cancers [7, 8]. Disruption in miRNA levels have been reported in numerous disease processes. So, they have the potential to be developed into novel therapeutic targets [9–11].

miR-206 is a member of the miR-1 family. The gene encoding this miRNA is located between the *IL-17* and *PKHD1* genes in human [5]. The cytogenetic band of miR-206 is 6p12.2. This miRNA has been shown to participate in the pathogenesis of a variety of malignant and non-malignant conditions. Like other member of this miRNA family, miR-206 has physiological roles as well. Members of mouse mir-1 family have important functions in muscle development [12]. In the current review, we summarize the role of miR-206 in both malignant and non-malignant conditions and explain its possible therapeutic implications.

## Bioinformatics step

### Prediction of miR-206 target genes

**Fig. 1:**
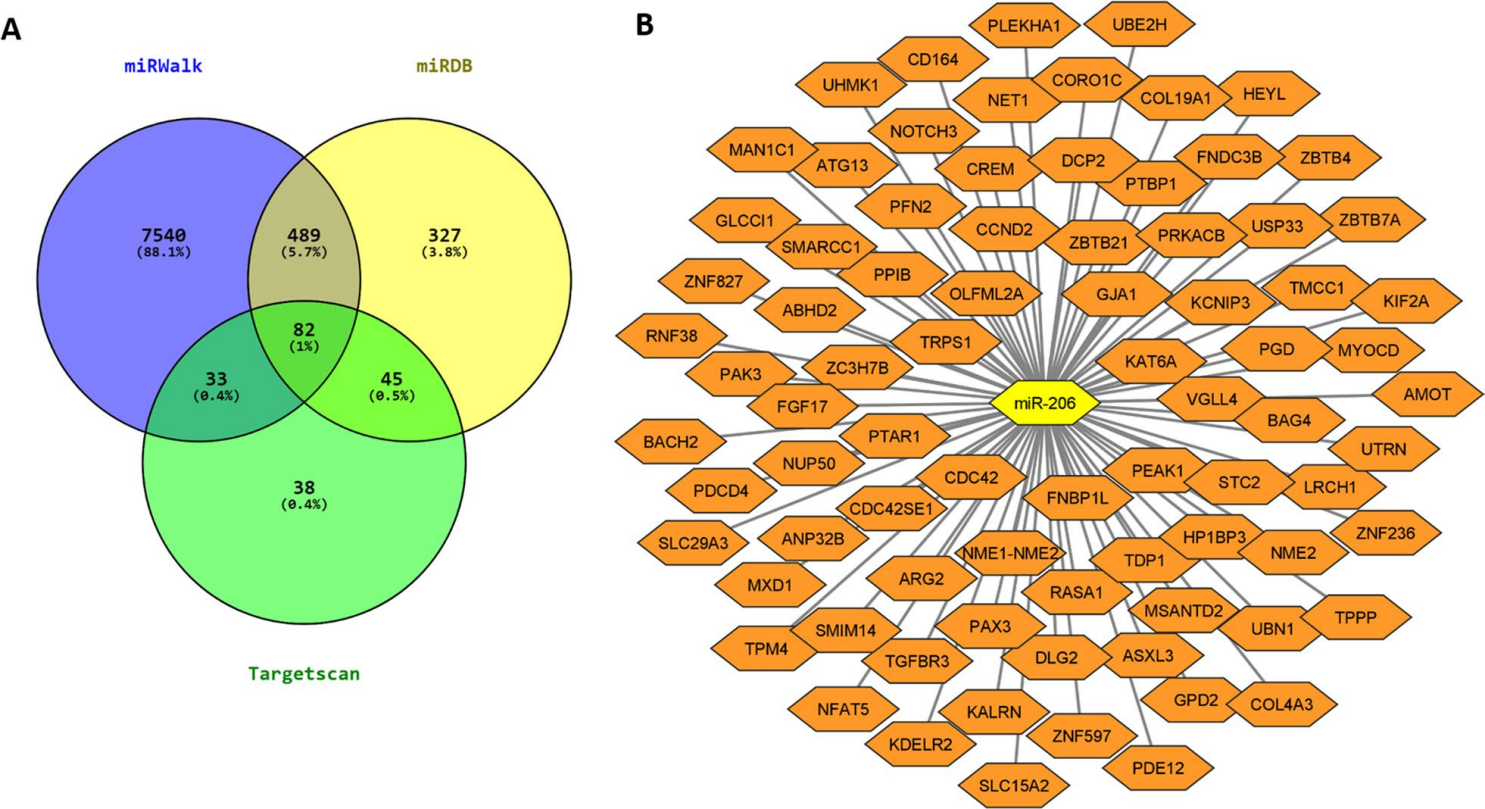
miR-206 target genes. **A** Prediction of a total of 82 common target genes in the three databases miRWalk, miRDB, and TargetScan. **B** 82 common genes targeted by miR-206. The interaction network was constructed by Cytoscape software (Cytoscape (3.9.1), using Java 11, Free Software Foundation, Inc., MA 02111–1307 USA)

miRWalk (http://mirwalk.umm.uni-heidelberg.de/), miRDB (http://www.mirdb.org/), and TargetScan databases (https://www.targetscan.org/) were used to predict the miR-206 target genes. A total of 82 mRNAs have been identified as common targets of this miRNA in these three databases (Fig. 1). Based on the results, miR-206 is predicted to target a variety of genes being involved in a wide range of cellular functions.

### Gene ontology (GO) and pathway enrichment analysis of miR-206 target genes

The Enrichr online database (https://maayanlab.cloud/Enrichr/) was used to perform GO analyses for the top miR-206 target genes. The target genes of miR-206 were remarkably enriched in the following GO terms: activation of cysteine-type endopeptidase activity involved in apoptotic process, collagen fibril organization, and hemopoiesis in biological process (BP), intracellular membrane-bounded organelle, actin cytoskeleton, and nucleus in cellular component (CC); and RNA polymerase binding, methyl-CpG binding, and DNA binding in molecular function (MF) (Table 1).

**Table 1:** Gene ontology enrichment analyses of top miR-206 target genes

GO Term	Category	Description	P-value	Genes
Biological process	(GO:0006919)	Activation of cysteine-type endopeptidase activity participating in apoptotic process	0.0035	COL4A3;ANP32B
	(GO:0030199)	Collagen fibril organization	0.0042	COL4A3;PPIB
	(GO:0030097)	Hemopoiesis	0.0047	TGFBR3;CD164
	(GO:1904428)	Negative regulation of tubulin deacetylation	0.0054	TPPP
	(GO:1905563)	Negative regulation of vascular endothelial cell proliferation	0.0054	COL4A3
	(GO:0014855)	Striated muscle cell proliferation	0.0054	TGFBR3
	(GO:0032277)	Negative regulation of gonadotropin secretion	0.0054	GJA1
	(GO:0051418)	Microtubule nucleation by microtubule organizing center	0.0054	TPPP
	(GO:00600	Cardiac muscle cell proliferation	0.0054	TGFBR3
	(GO:0034694)	Response to prostaglandin	0.0054	TGFBR3
Molecular function	(GO:0070063)	RNA polymerase binding	0.0024	ANP32B;PPIB
	(GO:0008327)	Methyl-CpG binding	0.0043	ZBTB21;ZBTB4
	(GO:0003677)	DNA binding	0.0059	KAT6A;TDP1;HP1BP3;ZBTB21;NME2;CREM;PAX3;ZBTB4;ZBTB7A
	(GO:0000978)	RNA polymerase II cis-regulatory region sequence-specific DNA binding	0.0070	NFAT5;HEYL;KCNIP3;CREM;PAX3;ZNF236;MXD1;ZBTB4;ZBTB7A;BACH2;ZNF597
	(GO:0000987)	Cis-regulatory region sequence-specific DNA binding	0.0070	NFAT5;HEYL;KCNIP3;CREM;PAX3;ZNF236;MXD1;ZBTB4;ZBTB7A;BACH2;ZNF597
	(GO:0016896)	Exoribonuclease activity, producing 5'-phosphomonoesters	0.0080	PDE12;DCP2
	(GO:0043565)	Sequence-specific DNA binding	0.0083	NFAT5;ZBTB21;CREM;PAX3;ZBTB4;ZBTB7A;BACH2;ZNF597
	(GO:0000977)	RNA polymerase II transcription regulatory region sequence-specific DNA binding	0.0091	NFAT5;HEYL;TRPS1;KCNIP3;CREM;PAX3;ZNF236;MXD1;ZBTB4;ZBTB7A;BACH2;ZNF597
	(GO:0019900)	Kinase binding	0.0116	CDC42;CCND2;DLG2;UTRN;ATG13;ZBTB4
	(GO:0003690)	Double-stranded DNA binding	0.0173	NFAT5;TDP1;CREM;PAX3;ZBTB7A;BACH2;ZNF597
Cellular component	(GO:0043231)	Intracellular membrane-bounded organelle	0.0015	NFAT5;HP1BP3;RNF38;ZBTB21;CREM;ZBTB4;PTBP1;GJA1;BAG4;CCND2;ZNF827;TRPS1;UBN1;TPPP;PRKACB;ZBTB7A;MYOCD;SMARCC1;UBE2H;NME2;PGD;UHMK1;HEYL;KAT6A;TDP1;COL4A3;PDCD4;KDELR2;ANP32B;ZNF236;PPIB;VGLL4;ZNF597;SLC29A3
	(GO:0015629)	Actin cytoskeleton	0.0018	NOTCH3;TPM4;PEAK1;KALRN;AMOT;CORO1C
	(GO:0005634)	Nucleus	0.0024	NFAT5;HP1BP3;RNF38;ZBTB21;CREM;ZBTB4;PTBP1;BAG4;CCND2;ZNF827;TRPS1;UBN1;TPPP;PRKACB;ZBTB7A;MYOCD;SMARCC1;UBE2H;NME2;PGD;UHMK1;HEYL;KAT6A;TDP1;PDCD4;ANP32B;ZNF236;PPIB;VGLL4;ZNF597
	(GO:0030660)	Golgi-associated vesicle membrane	0.0043	GJA1;KDELR2
	(GO:0070160)	Tight junction	0.0051	GJA1;UBN1;AMOT
	(GO:0016600)	Flotillin complex	0.0203	CORO1C
	(GO:0099513	Polymeric cytoskeletal fiber)	0.0210	TPM4;KIF2A;TPPP;AMOT
	(GO:0030175)	Filopodium	0.0236	CDC42;UTRN
	(GO:0005587)	Collagen type IV trimer	0.0243	COL4A3
	(GO:0030137)	COPI-coated vesicle	0.0283	KDELR2

**Fig. 2:**
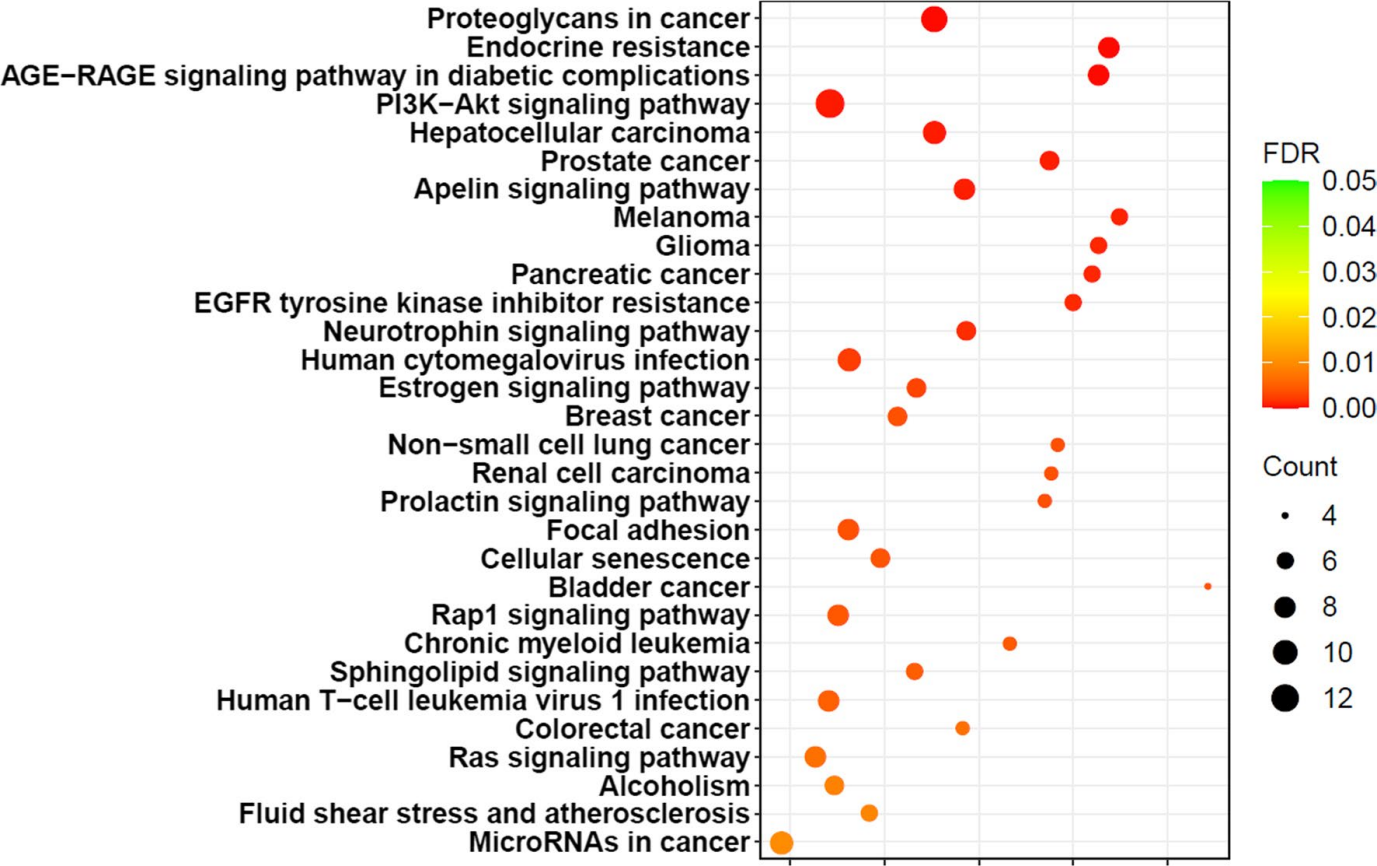
Bubble Plot of the Top 30 Enriched KEGG Pathways for miR-206, using Cancer MIRNome database

Additionally, the Kyoto Encyclopedia of Genes and Genomes (KEGG) pathway enrichment analyses were conducted using CancerMIRNome database (http://bioinfo.jialab-ucr.org/CancerMIRNome/). This uncovered that Proteoglycans in cancer, Endocrine resistance, and AGE-RAGE signaling pathways were the top-ranked pathways for miR-206 (Fig. 2).

### Literature search step

#### Role of miR-206 in cancers

**Fig. 3:**
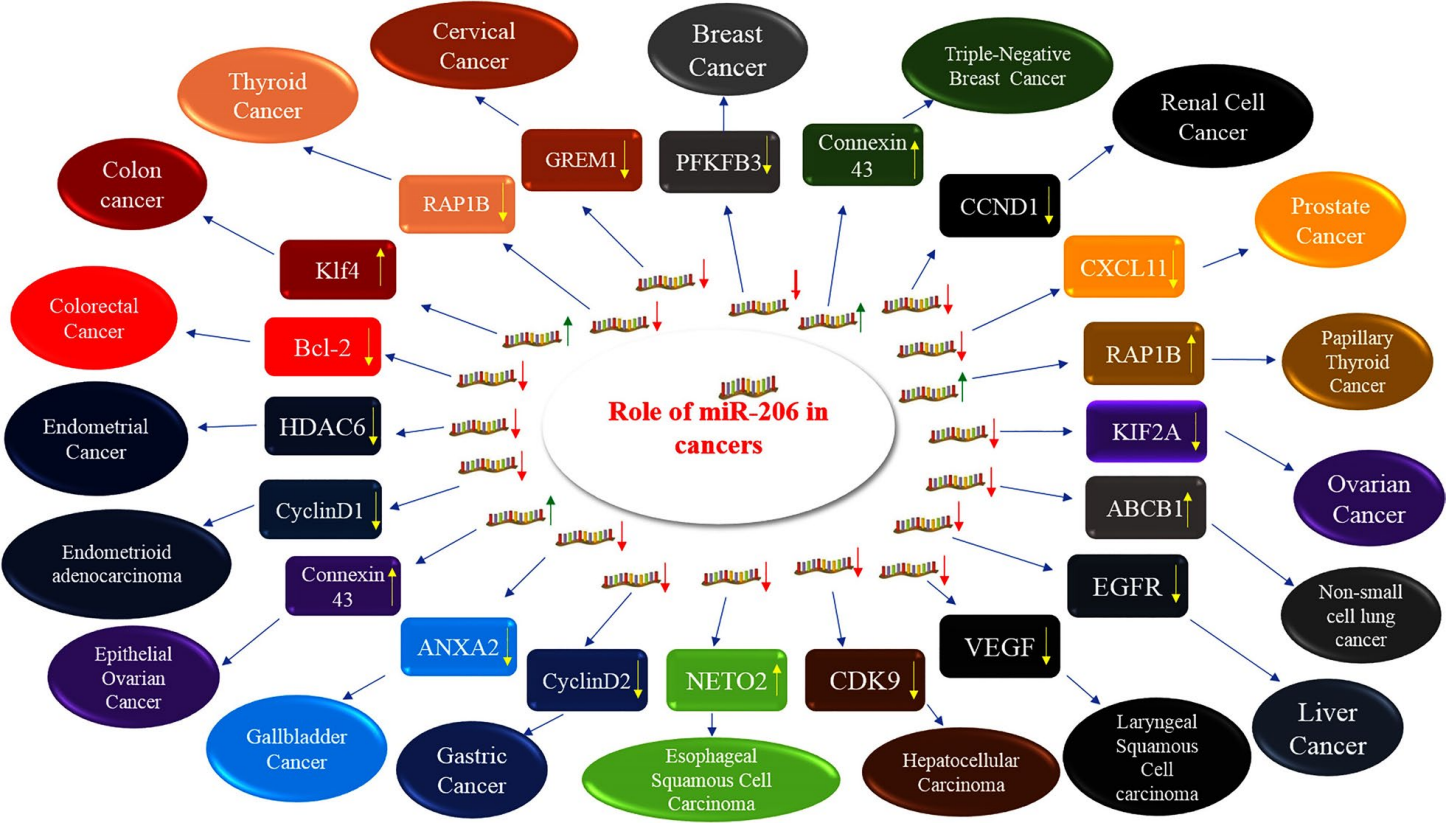
Effect of miR-206 in human cancers. miR-206 exerts its role in the development of cancer through modulation of expression of a variety of targets. Detailed data is shown in Table 2

Several studies have assessed expression of miR-206 in different types of cancers and found the molecular mechanism of involvement of this miRNA in the carcinogenesis (Fig. 3).

An experiment in bladder cancer tissues has shown over-expression of lncRNA RMRP in these tissues compared with adjacent tissues using qRT-PCR method. As revealed by MTT assay and transwell assay, RMRP induces cell proliferation, migration and invasiveness of bladder cancer cells through regulation of miR-206. The latter finding is based on the observed binding of miR-206 and RMRP in luciferase assay [13].

Similarly, in breast cancer cells, miR-206 has a tumor suppressor role possibly through down-regulation of PFKFB3. Expression of miR-206 in estrogen receptor α (ERα) positive breast cancer cells has been found to be reduced by 17β-estradiol in a dose-dependent manner. Over-expression of miR-206 could impede production of fructose-2,6-bisphosphate, diminish lactate synthesis and reduce proliferative ability and migration of breast cancer cells [14]. An independent study in breast cancer has shown association between down-regulation of miR-206 and large tumor dimension and advanced clinical stage. Over-expression of miR-206 in MCF-7 cells has suppressed cell growth through hindering G1/S transition. This effect is mediated through suppression of expression of cyclin D2. Consistent with this finding, expression levels of miR-206 have been inversely correlated with those of cyclin D2 in breast cancer tissues [15]. Expression of miR-206 has also been shown to be reduced in ERα-positive breast tumors. Besides, expression of miR-206 has been inversely related with ERα but not ERβ transcript levels in breast cancer tissues. Forced over-expression of miR-206 into MCF-7 cells has led to reduction of cell growth in both dose- and time-dependent manners, implying that miR-206 can be a target for endocrine therapy in this type of cancer [16]. Similarly, miR-206 has been shown to inhibit stemness and metastastatic ability of breast cancer cells through influencing activity of MKL1/IL11 axis [17]. Moreover, this miRNA can suppress epithelial mesenchymal transition (EMT) through influencing activity of TGF-β signals in ER-positive breast cancer cells [18].

miR-206 has also been down-regulated in the cervical cancer tissues, parallel with up-regulation of its target gene c-Met as revealed by qRT-PCR assay and immunohistochemistry. Kaplan–Meier and log-rank analyses have shown relation between down-regulation of miR-206 and shorter overall survival. Besides, down-regulation of miR-206 in cervical cancer tissues has been associated with lymph node metastasis, advanced stage and advanced histological grade indicating the role of miR-206 in the metastasis and progression of cervical cancer. In fact, miR-206 has been found to be independent prognostic marker for overall survival of patients with this type of cancer [19]. Table 2 shows summary of the role of miR-206 in malignant conditions.

**Table 2:** Summary of the role of miR-206 in malignant conditions (PTANTs: pairs of primary tumor tissues and adjacent normal tissues)

Type of cancer	Expression Pattern	Samples	Animal studies/cell lines	Downstream targets	Pathway	Function	Kaplan–Meier	Refs.
Bladder Cancer (BC)	Down-regulation (transfected with RMRP promoter)	20 serum specimens of BC patients	SV-HUC-1, BIU-87 and T24	–	–	LncRNA RMRP acts as a miR-206 sponge and promotes proliferation, migratory aptitude and invasion in BC	–	[13]
Breast cancer	Down-regulation	59 PTANTs	MCF-7, T47D and SUM159	PFKFB3	Pentose phosphate pathway (PPP)	miR-206 reduces viability, proliferation and migration. miR-206 overexpression moderates glycolysis through PFKFB3 suppression	–	[14]
	Down-regulation	Breast cancer tissues and normal tissues	HEK293T, MCF-7	CyclinD2	–	miR-206 suppresses proliferation and colony formation through hindering G1/S transition. Downregulation of miR-206 is associated with larger tumor size and advanced clinical stage	–	[15]
	Down-regulation (In estrogen receptor (ER) -positive compared with ERa-negative)	94 breast tumor tissues	MCF-7	–	–	miR-206 suppresses ERa expression and inhibits cell growth	–	[16]
	Down-regulation	GEO-GSE 59,751	MDA-MB-231, MCF-7, HS578t, and Hek293T Xenograft models	TWF1, IL11, MKL1 and SRF	MKL1/SRF	miR-206 inhibited cell cycle and self-renewal in tumorigenesis. It hampered cell motility	–	[17]
	Down-regulation (in MCF-7 and MDA-MB-231 cell lines in compared with MCF-10A)	miRaNda algorithm, MiRmap and TargetScan	MCF-7, MDA-MB-231, MCF-10A and HEK-293 T	NAMPT	Nicotinamide Adenine Dinucleotide (NAD)	miR-206 suppresses cell survival and induced cell apoptosis through regulating NAMPT. Also, it plays a rule in breast cancer cell growth regulation	–	[20]
	Down-regulation	–	MDA-MB-231 and MCF-7	GATA-3, ER_, SRC-1, and SRC-3	EGF/EGFR, EGFR/MAPK	miR-206 attenuates cell proliferation, induces apoptosis, and decreased expression of several estrogen-responsive genes by EGFR signaling	–	[21]
	Down-regulation	–	MCF-7 and T47D	neuropilin-1 (NRP1), SMAD2, phospholipase D1 (PLD1)	TGF-β	Up-regulation of miR-206 prohibited migration and invasion of ER positive breast cancer cell lines. It also repressed EMT	–	[18]
	Down-regulation	15 paclitaxel-sensitive patients and 15 paclitaxel-resistant patients	MCF-7, MDA-MB-231, MDA-MB-468, MDA-MB-453 and MCF-10 male BALB/c nude mice	FTH1P3, ABCB1	FTH1P3/miR-206/ABCB1	miR‐206 sponged by FTH1P3 in paclitaxel‐resistant patients and provided a novel insight in breast cancer chemoresistance	–	[22]
	Up-regulation	82 breast tumor tissues and their adjacent normal tissues	MCF10A, MDA-MB-231, SK-BR-3 Female BALB/c-nu mice	NK1R-FL, Erk1/2	Phosphatidylinositol pathway, MAPK	miR-206 promotes invasion, proliferation, colony formation and migration in human breast cancer cells	–	[23]
Cervical cancer	Down-regulation	41 PTANTs	–	Bcl2 and c-Met	–	Down-regulation of miR-206 induced advanced stage, advanced histological grade, metastasis and shorter survival in cervical cancer	Shorter overall survival	[19]
	Down-regulation	35 PTANTs	Ect1/E6E7, Caski, C33A, SiHa and HeLa	GREM1	Circ_0007534/miR-206/GREM1	miR-206 inhibited the progression of cervical cancer by downregulation of GREM1	–	[24]
	Down-regulation	50 cervical cancer tumor tissues	SiHa, HeLa and Normal human endocervical epithelial cells (NEECs)	BAG3, EGFR, MMP2, and MMP9	miR-206–BAG3	miR-206 suppresses proliferation, migration, and invasion	–	[25]
	Down-regulation	–	HeLa, mouse	Notch3	Notch3	miR-206 acts as a tumor suppressor and activates cell death	–	[26]
Colon cancer	Up-regulation	–	HCT116, HT29, Caco2, SW48, SW480 and CCD841 Male F344 rats	Klf4	–	Up-regulation of miR-206 plays a key role in etiology of cancers via targeting KLF4 and other pluripotency and cancer stem-cell factors. It also increased cell proliferation kinetics in colon cancer cell lines	–	[27]
Colorectal Cancer (CRC)	Down-regulation (in 5-FU-resistant cells)	–	HCT116 and RKO	Bcl-2	–	miR-206 regulated chemoresistance, proliferation, and apoptosis in CRC by targeting Bcl-2	–	[28]
Endometrial Cancer (EC)	Down-regulation	36 EC patients and 8 patients with dysfunctional uterine bleeding and normal curettage	AN3C, RL95 and HEC-1-A	HDAC6	PTEN/AKT/mTOR	miR-206 suppresses the proliferation, migration and invasion of endometrial cancer cells, via the PTEN/AKT/mTOR pathway	Poorer survival probability by targeting HDAC6	[29]
Endometrioid adenocarcinoma (EEC)	Down-regulation (in ERa-positive EECs)	30 fresh-frozen EEC tissue samples	RL95-2, Ishikawa and KLE	CyclinD1	MAPK	Expression of the tumor suppressor miR-206 is associated with inhibition of cell proliferation and reduces invasion in ERa-positive endometrioid adenocarcinoma	-	[30]
Epithelial Ovarian Cancer (EOC)	Down-regulation	50 EOC tissues and 20 normal tissues	SKOV3, HO8910, A2780, OVCAR, and HOSEpiC	c-Met	AKT/mTOR	miR-206 suppresses tumor cell growth, cell invasion and migration in EOC and down-regulation of miR-206 induced human EOC progression	Poor survival after surgery	[31]
	Up-regulation (in primary platinum-resistant EOCs)	56 EOC patients	OV2008, A2780, Twenty female SCID mice	Connexin43 (Cx43)	–	Up-regulation of miR-206 enhanced cell viability, migration and invasion in the presence of cisplatin and decreased cisplatin-induced apoptosis in cisplatin-sensitive EOC cell lines	Shorter overall survival	[32]
Esophageal Squamous Cell Carcinoma (ESCC)	Down-regulation	30 PTANTs	ECA109, TE-1, KYSE150, KYSE-410 and HET-1A	NETO2 and FOXP1	–	FAM225A increased NETO2 and FOXP1 levels by sponging miR-206 to advanced ESCC progression and angiogenesis	Shorter overall survival with a high level of FAM225A	[33]
Gallbladder Cancer (GBC)	Down-regulation	30 PTANTs	H69, NOZ, EH-GB1 GBC-SD and SGC-996	ANXA2 and KRAS	–	miR-206 inhibits ANXA2 and KRAS expression, which increase GBC progression	–	[34]
Gastric Cancer (GC)	Down-regulation	30 PTANTs	SGC-7901, MKN-28, MKN-45 and GES-1	CyclinD2 (CCND2)	–	miR-206 suppresses tumor cell growth, proliferation and induces cell cycle arrest at G0/G1 phase	–	[35]
Hepatocellular Carcinoma (HCC)	Down-regulation	–	HepG2, Bell740, HLE and L02	Cyclin-dependent kinase (CDK9) and Mcl-1	miR-206/CDK9	miR-206 suppresses tumor cell growth and proliferation by targeting CDK9	–	[36]
Laryngeal Squamous Cell Carcinoma (LSCC)	Down-regulation	35 LSCC patients	Hep-2, BALB/c mice	VEGF	VEGF	Downregulation of miR-206 increases the proliferation and invasion of LSCC	Poor survival	[37]
	Down-regulation	50 PTANTs	Hep-2, BALB/c nude mice(n = 12)	cyclinD2	–	miR-206 inhibited the growth and tumorigenicity of LSCC cells via repressing cyclin D2 expression	–	[38]
	Down-regulation	311 LSCC and adjacent non‐tumorous tissues	TU-212 cells, AMC-HN-8 cells and HEK-293 T, BALB/c nude mice (n = 18)	DNMT3A	–	RP11-159K7.2 sponged miR-206 and thus, increased LSCC cell proliferation and invasion	Poorer overall survival in low expression of miR-206	[39]
Liver Cancer	Down-regulation	HCC patients’ tissue samples	HL7702 (L02), Huh7, HepG2, Hep3B, CSQT-2, PLC and HCCLM3	EGFR	EGFR	miR-206 suppressed the expansion of liver cancer stem cells via regulating EGFR	–	[40]
Lung Cancer	Down-regulation (in high-metastatic strain)	35 patients (20 adenocarcinoma patients, 15 squamous carcinoma patients)	95D, 95C, A549, 801D, male BALB/c nude mice(n = 10)	MET	–	miR-206 inhibited cell migration, invasion and metastasis	–	[41]
	Down-regulation	–	PC-9, HCC827, male BALB/c nude mice(n = 9)	EGFR, c-Met	c-Met-Akt/Erk and Erk1/2	miR-206 can restore HGF-induced gefitinib resistance in EGFR activated lung cancer cells by inhibition of Akt/Erk pathways and EMT	–	[42]
Nasopharyngeal carcinoma (NPC)	Down-regulation (in CNE2-IR compared with CNE2 cells)	–	CNE2, CNE2-IR	IGF1	PI3K/AKT	miR-206 intenerates NPC cell to irradiation through targeting IGF1	–	[43]
Non-small cell lung cancer (NSCLC)	Down-regulation (in gefitinib-resistant NSCLC tumor tissues)	78 lung cancer tissues (Gefitinib resistant (n = 36) and Gefitinib-sensitive (n = 42))	PC9, Gefitinib-resistant PC9 (PC9/GR), Female BALB/C nude mice (n = 12)	ABCB1	SNHG14-miR-206-3p-ABCB1	miR-206-3p contribute to the chemoresistance of NSCLC to gefitinib via increasing ABCB1	–	[44]
Ovarian cancer	Down-regulation	108 human ovarian cancer tissue samples	A2780, SKOV3	KIF2A	–	Up-regulation of miR-206 enhanced apoptosis and inhibited proliferation, migration and invasion via decreasing KIF2A	–	[45]
	Down-regulation	35 ovarian cancer cases and 17 normal cases	SKOV3, ES2 and OVCAR3, Female BALB/c nude mice (n = 10)	TBX3	–	HOTAIR increases TBX3 expression via targeting miR-206, and promoting ovarian cancer stem cells	–	[46]
Papillary thyroid cancer (PTC)	Down-regulation	23 patients with PTC	Nthy-ori3-1, TPC-1, TPC-1/euthyrox	MAP4K3	MAPK, p38 and JNK	Up-regulation of miR-206 attenuated chemoresistance of drug-resistant PTC cells	–	[47]
	Up-regulation (in-baicalein treated patients)	–	TPC-1	RAP1B	miR-206/RAP1B	miR-206 is involved in baicalein inhibition of PTC cell growth by regulating miR-206/RAP1B pathway	–	[48]
Prostate cancer (PCa)	Down-regulation	35 patients with prostate cancer	22RV1, DU145, PC3, and 293 T, female BALB/c nude mice (n = 24)	ubiquitin-specific peptidase 33 (USP33)	–	circ_0057558 sponged miR-206 and increased cell proliferation and cell cycle transition in prostate cancer cell lines	Longer overall survival	[49]
	Down-regulation	10 pairs of PCa with adjacent control tissues	PC-3, DU-145, LNCaP and RWPE-1	CXCL11	–	miR-206 suppressed proliferation, tumor growth and activated cellular migration and invasion	–	[50]
Renal cell cancer (RCC)	Down-regulation	42 RCC specimens	786-O, OS-RC-2, and HK-2	G-associated kinase (GAK)	–	miR-206 acts as a tumor suppressor through targeting GAK	–	[51]
	Down-regulation	18 clear cell renal cell carcinoma (ccRCC) and 8 patients with benign renal tumors (BRT) and validation cohort (68 ccRCC, 47 BRT, and 28 healthy cases)	–	CDK4, CDK9, and CCND1	–	Up-regulation of miR-206 inhibited cell proliferation and colony formation	Shorter overall survival and progression-free survival	[52]
Rhabdomyosarcoma (RMS)	Up-regulation	10 patients with RMS, 28 patients with other pediatric tumors and 17 healthy cases	Rh30, SCMC-RM2, RD RMS-YM, CT-TC, Rh18 and Rh41, IMR32, GOTO, SK-N-SH, KP-N-RT, KP-EWS-YI, KPEWS-AK and KP-EWS-G401 and MRT-YM	–	–	miR-206 has the highest specificity and sensitivity among muscle-specific miRNAs, so it is the best biomarker for RMS prediction	–	[53]
Head and neck squamous cell carcinoma (HNSCC)	Down-regulation	22 pairs of primary tumors and normal epithelial samples, and 23 formalin-fixed paraffin-embedded tissues	FaDu, SAS and HSC3	EGFR, c-MET, AKT and ERK1/2	MAPK, Actin cytoskeleton and ECM–receptor, focal adhesion	Downregulation of miR-206 induced cancer cell aggressiveness via targeting EGFR and c-MET in HNSCC cells	–	[54]
Thyroid cancer (TC)	Down-regulation	60 tumor samples and matched noncancerous specimens	8505C,TPC-1, SW1736, SW579 and Nthy-ori 3–1, female NOD/SCID mice (n = 10)	RAP1B	protein kinase A (PKA)	miR-206 inhibited cell activities of proliferation, invasion, and migration in TC via suppressing RAP1B expression	–	[55]
Triple-negative breast cancer (TNBC)	Down-regulation	–	MDA-MB-231 and MDA-MB-436 Female athymic (nu/nu) BALB/c mice	Connexin43 (Cx43)	–	miR-206 represses the proliferation and invasion of TNBCs		[56]
	Down-regulation	83 TNBC tissues and 124 normal breast tissue samples	MCF-10A, MDA-MB-231, MDA-MB-468, SK-BR-3 and MCF-7, TNBC-bearing mice	–	PI3K/AKT/mTOR	miR-206 inhibited proliferation, migration, invasion, chemo-sensitivity and autophagy of TNBC cells	Poor 3-years survival	[57]
	Down-regulation	24 primary tumors and 13 normal breast tissue samples	MDA-MB-231, SUM159, MCF-10A, MCF-7 and T47D Mouse HC11	CORO1C, TMSB4X, TPM4, and TNS3	–	miR-206 reduced proliferation and migration in TNBC patients		[58]

Diagnostic role of miR-206 has been assessed in epithelial ovarian cancer [32], renal cell carcinoma [52] and rhabdomyosarcoma [53] (Table 3). In epithelial ovarian cancer, miR-206 levels can be used for discrimination of patients with incomplete response to platinum chemotherapy from those with complete response to this modality with area under the receiver characteristic curve (AUC) of 0.82 [32]. Most notably, expression levels of miR-206 has a high accuracy in discrimination of patients with rhabdomyosarcoma from healthy subjects with AUC value of 0.96 [53].

**Table 3:** Diagnostic value of miR-206 in cancers

Type of disease	Number of samples	Distinguish between	Area under curve	Sensitivity (%)	Specificity (%)	Refs.
Epithelial ovarian cancer (EOC)	56 EOC patients	27 incomplete response (IR) EOC patients vs. 29 complete response (CR) patients	0.829	–	–	[32]
Renal cell cancer (RCC)	18 ccRCC and 8 patients with benign renal tumors (BRT); validation cohort: 68 ccRCC, 47 BRT, and 28 healthy cases	ccRCC patients vs. healthy controls	0.733	83.8	57.1	[52]
Rhabdomyosarcoma (RMS)	10 patients with RMS, 28 patients with other pediatric tumors and 17 healthy cases	Pediatric tumor patients vs. healthy volunteers	0.967	100	91.3	[53]

#### Role of miR-206 in non-malignant conditions

**Fig. 4:**
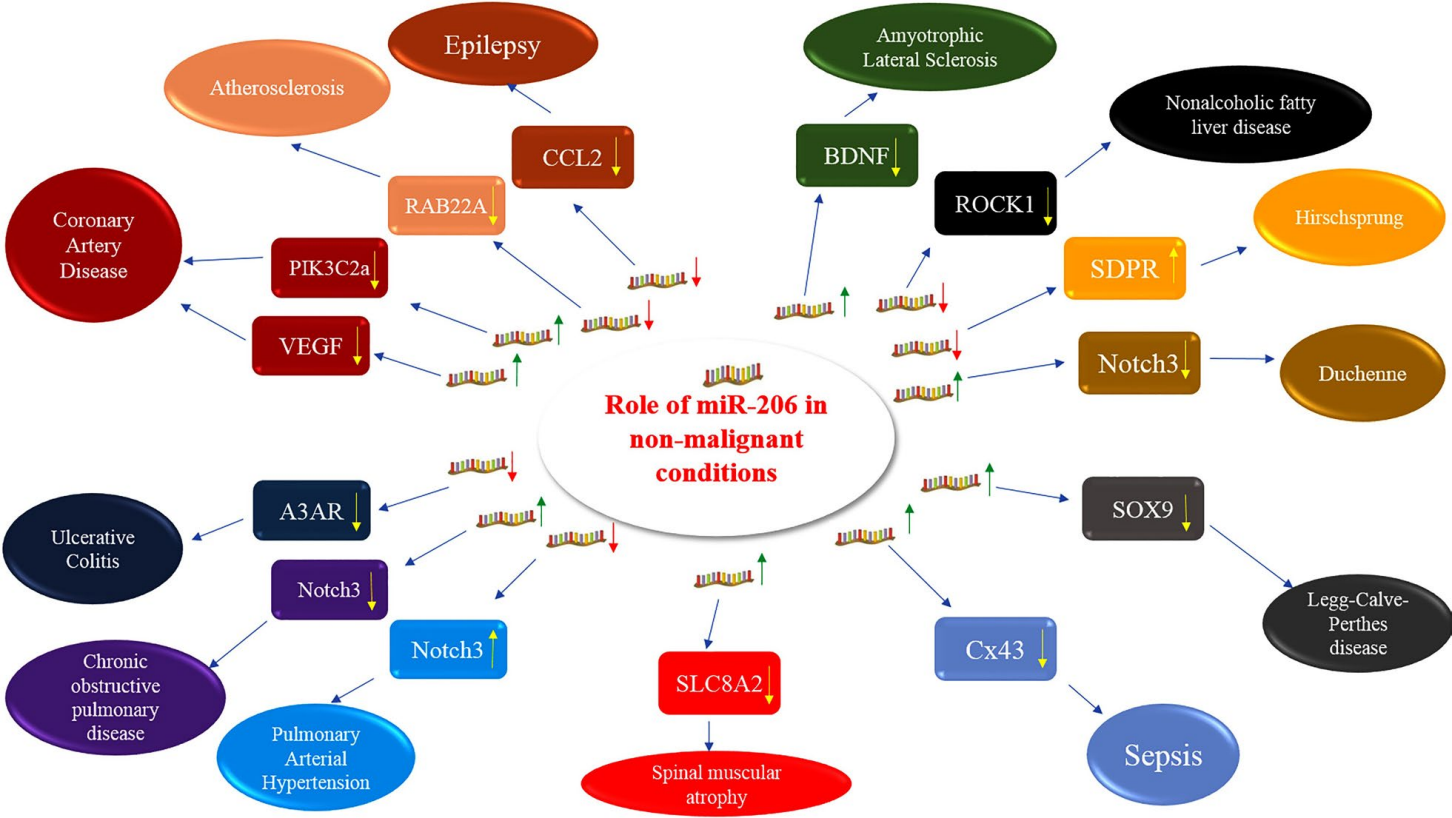
Role of miR-206 in non-malignant disorders. Detailed data about experiments is shown in Table 4

miR-206 has crucial effects in the pathophysiology of several non-malignant disorders through modulation of a variety of targets (Fig. 4).

Expression assays in amyotrophic lateral sclerosis (ALS) have shown dysregulation of circulatory levels of several miRNAs in these patients. Notably, miR-206 has been among up-regulated miRNAs in these patients. In addition, constant changes in miRNAs signature have been found to persist during progression of ALS. This finding indicates the potential of selected miRNAs such as miR-206 as longitudinal markers for this disorder [59].

Circulatory levels of a number of miRNAs such as miR-206 have also been used as possible predictors for the progression of amnestic mild cognitive impairment (aMCI) to Alzheimer's disease (AD). Notably, serum levels of miR-206 have been found to be higher in aMCI patients progressed to AD. Kaplan–Meier analysis has also demonstrated remarkable correlation between conversion of aMCI to AD and over-expression of miR-206 [60]. Over-expression of miR-206 in olfactory mucosal cells can also been used as an early diagnostic approach in AD [61]. Another experiment in animal models of AD and temporal cortex samples from AD patients has verified over-expression of miR-206. These effects are mainly mediated through modulation of BDNF expression. In fact, a neutralizing inhibitor of this miRNA could prevent the harmful effect of amyloid-β42 on BDNF and dendritic spine degeneration [62]. On the other hand, another study has shown a neuroprotective effect of miR-206-3p in AD [63]. Table 4 shows summary of the role of miR-206 in non-malignant conditions.

**Table 4:** Summary of the role of miR-206 in non-malignant conditions

Type of disease	Expression Pattern	Samples	Animal studies/cell lines	Downstream targets	Pathway	Function	Kaplan–Meier	Refs.
Amyotrophic Lateral Sclerosis (ALS)	Up-regulation	27 sporadic ALS patients and 25 control subjects	–	–	–	miR-206 contributes in ALS progression, so act as a longitudinal biomarker	–	[59]
Alzheimer’s disease (AD)	Up-regulation	128 subjects with amnestic mild cognitive impairment (aMCI) who progressed to AD (aMCI-AD) and 330 subjects who maintained and aMCI (aMCI- aMCI) diagnosis	–	Brain-derived neurotrophic factor (BDNF)	–	miR-206 plays a key role in the progression of aMCI to AD by targeting BDNF	A significant AD conversion trend for aMCI patients having high levels of miR-206	[60]
	Up-regulation	24 olfactory epithelia of early dementia patients, 8 Patients with significant depression and 9 cognitively healthy controls	–	BDNF	–	miR-206 increases from the MCI stage, and shows excellent sensitivity and specificity for diagnosing CDR1 dementia	–	[61]
	Up-regulation	–	Tg2576 AD transgenic mice, Neuro-2a	BDNF	–	miR-206 contributes in the pathogenesis of AD by suppressing BDNF expression	–	[62]
	–	–	36 C57 mice	BDNF	–	miR-206-3p protects neurons via up- regulation of BDNF after the onset of AD. Exogenous miR-206-3p further ameliorates the neuronal morphology, and improves the cognitive ability and memory of AD mice	–	[63]
	Up-regulation	–	SHSy5y, APP/PS1 mice	BDNF	–	miR-206 induces cell death via downregulating the expression of BDNF	–	[64]
Atherosclerosis (AS)	Down-regulation (treated with ox-LDL)	–	HUVECs and 293 T	RAB22A	–	MIAT targeted miR-206 and promoted cell viability, invasion, migration, and EMT	–	[65]
Bronchopulmonary dysplasia (BPD)	Down-regulation	–	AECII, old Sprague–Dawley female rats	–	–	miR-206 inhibits FN1 expression and proliferation	–	[66]
Coronary Artery Disease (CAD)	Up-regulation	78 CAD patients and 65 healthy cases	–	vascular endothelial growth factor (VEGF)	VEGF	miR-206 inhibited cell viability and invasion, promoted apoptosis, and has some protective roles in CAD	–	[67]
	Up-regulation	53 Peripheral blood mononuclear cells CAD and 34 healthy cases	Nude mice	PIK3C2a	PI3K/Akt/eNOS	miR-206 downregulated angiogenesis by repression of the PI3K/Akt/eNOS signaling	–	[68]
Chronic obstructive pulmonary disease (COPD)	Up-regulation	44 tissue samples of patients	HPMECs	Notch3, and VEGFA	Notch and VEGFA	Up-regulation of miR-206 promoted cell apoptosis by repressing Notch3, and VEGFA	–	[69]
Duchene muscular dystrophy (DMD)	Up-regulation	–	Mdx mice	Notch3, Igfbp5	–	miR -206 induced satellite cell differentiation into muscle fibers through inhibiting negative myogenesis regulators. miR-206 slows progression of DMD	–	[70]
	Up-regulation	39 DMD patients and 36 healthy controls	–	–	–	miR-206, related to low muscle strength, muscle function, and quality of life	–	[71]
Epilepsy	Down-regulation	–	Male Sprague–Dawley rats	C–C Motif Chemokine Ligand 2 (CCL2)	–	miR-206 suppresses epilepsy and seizure-induced brain damage through targeting CCL2	–	[72]
Non-alcoholic fatty liver disease (NAFLD)	Down-regulation	–	24 high-fat diet (HFD) feeding mouse, Huh-7 and HepG2	ROCK1	ROCK1/AMPK	miR-206 inhibited lipogenesis via targeting ROCK1 and repressed triglyceride secretion which contributed to NAFLD development and progression	–	[73]
Hirschsprung (HSCR)	Down-regulation	80 HSCR cases and 80 matched controls	293 T and SH-SY5Y	SDPR (serum deprivation response), FN1(fibronectin 1) and PAX3(paired box 3)	–	miR-206 inhibited cell migration and proliferation by up-regulation of SDPR	–	[74]
Legg-Calvé-Perthes disease (LCPD)	Up-regulation	20 LSPD tissue and 20 normal patients with repair surgery after fracture	TC28	SOX9	–	Up-regulation of miR-206 promoted cell apoptosis by repressing SOX9	–	[75]
Limb-girdle muscular dystrophies (LGMD)	Up-regulation	11 LGMD patients	–	–	–	Up-regulation of miR-206 occurs during skeletal muscle regeneration	–	[76]
Muscular dystrophies	Up-regulation (in BMD and DMD patient)	48 patients with DMD, DM1, LGMD, LGMD2B, FSHD, BMD, and DMRV and healthy controls	C57Bl/10SnSlc mice, mdx mice	–	–	miR-206 levels in mouse serum are up-regulated upon skeletal muscle regeneration	–	[77]
Pulmonary arterial hypertension (PAH)	Down-regulation	–	HPASMCs,mice	Notch3		Up-regulation of miR-206 inhibited migration, proliferation, contraction and enhanced apoptosis in PASMC’s of hypoxia induced PAH mice	–	[78]
Sepsis	Up-regulation	63 blood samples of Sepsis and 30 Septic Shock and 28 healthy controls	–	Cx43	–	miR-206 regulates the barrier function of ATII cells in sepsis-related acute lung injury through regulating expression of Cx43	–	[79]
Spinal muscular atrophy (SMA)	Up-regulation	–	80 WT mice and 74 SMA mice, PC12	SLC8A2	–	miR-206 decreases severity of SMA pathology, progression, increases survival rate and improved behavioral performance of mice	–	[80]
Ulcerative colitis (UC)	Down-regulation (In mesalamine-treated patients)	10 established UC patients	HT29	A3 adenosine receptor (A3AR)	–	miR-206 acts as a pro-inflammatory factor through direct suppression of A3AR expression	–	[81]

Diagnostic value of miR-206 has been evaluated in AD and muscular dystrophies (Table 5). In Duchene muscular dystrophy, expression levels of miR-206 can be used as a diagnostic marker with AUC value of 0.96 [71]. Similarly, this miRNA can be used as a marker for diagnosis of Becker muscular dystrophy [77].

**Table 5:** Diagnostic value of miR-206 in non-malignant diseases

Type of disease	Number of samples	Distinguish between	Area under curve	Sensitivity (%)	Specificity (%)	Ref.
Alzheimer’s disease (AD)	128 subjects with amnestic mild cognitive impairment (aMCI) who progressed to AD (aMCI-AD) and 330 subjects who maintained an aMCI (aMCI- aMCI) diagnosis	aMCI-AD subjects vs. aMCI- aMCI subjects	0.95	95.3	77.8	[60]
	24 olfactory epithelia of early dementia patients, 8 patients with significant depression and 9 cognitively healthy controls	Mild cognitive impairment group (CDR 0.5; n = 13) vs. all subjects CDR 1 group (n = 11) vs. all subjects	0.942 0.976	87.5 90.9	94.1 93.3	[61]
Duchene muscular dystrophy	39 DMD patients and 36 healthy controls	DMD vs. healthy controls	0.96	94	95	[71]
Muscular dystrophies	48 patients with DMD, DM1, LGMD, LGMD2B, FSHD, BMD, and DMRV and healthy controls	BMD vs. healthy controls	0.90	–	–	[77]

## Discussion

miR-206 is an example of miRNAs with crucial roles in the pathogenesis of a wide range of human disorders. In the context of cancer, expression assays using qRT-PCR method and functional studies have led to the supposition of miR-206 as a tumor suppressor miRNA (summarized in Table 2), although some exceptions have been demonstrated [53]. It can reduce proliferation of cancer cells and induce their apoptosis [14] via different routes. Moreover, it can regulate cell cycle progression through modulation of expression of cell cycle-related genes [15]. The activity of several oncogenic pathways is modulated by miR-206. Examples of these pathways are EGF/EGFR [21], EGFR/MAPK [21], TGF-β [18], Notch3 [26], PTEN/AKT/mTOR [29], VEGF [37], c-Met-Akt/Erk [42], Erk1/2 [42] and PI3K/AKT [43]. Most importantly, expression assays in patients with different responses to chemotherapeutic agents and functional studies in cell lines have shown that miR-206 can enhance cytotoxic effects of anti-cancer agents on cancer cells [22]. The latter finding highlights the importance of this miRNA in design of novel modalities to combat chemoresistance.

Among non-malignant conditions, dysregulation of miR-206 has been reported in amyotrophic lateral sclerosis, Alzheimer’s disease, atherosclerosis, bronchopulmonary dysplasia, coronary artery disease, chronic obstructive pulmonary disease, epilepsy, nonalcoholic fatty liver disease, Hirschsprung disease, muscular dystrophies, pulmonary arterial hypertension, sepsis and ulcerative colitis (summarized in Table 4). Thus, this miRNA can affect pathogenesis of a wide array of human disorders.

In silico studies have revealed that miR-206 can affect expression of tens of mRNAs being involved in the regulation of crucial cellular mechanisms such as activation of cysteine-type endopeptidase activity involved in apoptotic process, collagen fibril organization, hemopoiesis, regulation of tubulin deacetylation, regulation of vascular endothelial cell proliferation, regulation of gonadotropin secretion, response to prostaglandin, RNA polymerase binding, methyl-CpG binding and sequence-specific DNA binding. Therefore, it is not surprising that miR-206 influences pathoetiology of several disorders.

Notably, expression levels of miR-206 not only can be used for cancer diagnosis [53] and in determination of response to anti-cancer therapies [32], but also may be potential markers for discrimination of patients with muscular dystrophies from healthy subjects [71] or prediction of course of Alzheimer’s disease [61]. Since miRNAs can be easily tracked in the biofluids, these findings open a new era for detection of human disorders via non-invasive tools.

## Conclusion

Altered expression of miR-206 in tumor tissues has been associated with malignant characteristics of cancers in terms of higher metastatic aptitude and lower survival rate, implying the role of this miRNA as a prognostic marker. Finally, forced over-expression of miR-206 in many cancer cell lines has led to reduction of malignant characteristics in cell line assays as well as animal models. Thus, this strategy can be used as a novel therapeutic approach for cancers. Meanwhile, miR-206 is involved in the pathophysiology of several non-malignant conditions, including neurodegenerative and neuropsychiatric disorders and muscular atrophies. Since efficient therapies for these kinds of disorders have not been developed yet, miR-206-targetted therapies might revolutionize this research field.

## Data Availability

The analyzed data sets generated during the study are available from the co‑responding author on reasonable request.
